# The Legal Position of a Suspected Milk Supply

**Published:** 1905-07-08

**Authors:** 


### The Legal Position of a Suspected Milk Supply.

In his annual report for 1904, Dr. W. S. Cook, medical officer of health for the burgh of Greenock, relates an interesting discussion which recently arose in connection with an outbreak of enteric fever, and which in the end came into the law courts and was settled by a judicial decision. It appears that during September and October one hundred cases of enteric fever were notified from one and the same district of the town, and it was found that in all the cases the milk supply of the household was derived from an identical source. As the farm in question was outside the burgh the attention of the medical officer of health for the county was directed to the facts at the commencement of the epidemic, and later the burgh health authority formally notified the executive committee of the county with a view to the prohibition of the suspected milk supply. The county authorities, however, declined to take any action in this direction. In these circumstances the burgh authority appealed against this decision, and, after hearing the parties concerned, the sheriff sustained the appeal and ordered the supply of milk from the incriminated farm to be stopped, "until the further orders of the court."

In a note issued with his decision the sheriff discusses several points which are of considerable interest and importance to all those concerned in the administration of the laws relating to the public health. Regarding his own position and function in reference to such an appeal, the sheriff concludes that his duty is not to conduct an independent inquiry but to review the finding of the local health authorities on the facts submitted to them. That finding is a judicial rather than an administrative act. It is pronounced after considering reports and, perhaps also, other evidence, and the duty of the sheriff is to subject it to official review. In this particular instance the evidence consisted of a certificate from the medical officer that the delivery of milk from this farm was likely to spread enteric fever through the town of Greenock, and the fact that all, or nearly all, the infected persons received their milk supply from the farm in question. On the other hand, it was urged that the water-supply of the farm was from a source not liable to contamination; that all the cases of enteric had occurred in one and the same neighbourhood, and might, therefore, be presumed to have a local causation; that a number of customers of the farm outside this area had not acquired enteric; and, lastly, that there had been no recent illness at the farm. In reviewing these facts the sheriff held that it is not necessary to *prove* that a particular milk supply is the cause of an outbreak of infectious disease before a closing order is pronounced. The position is not comparable to an action for damages or to a criminal charge where the law requires that the relation between the two events as cause and effect shall be clearly established.

The object is not to remedy past, but to obviate the risk of future injury, and it is sufficient to show that there is reasonable ground for believing that contamination proceeds from a certain source though the evidence stops short of a clear demonstration that this is actually the case. Such a conclusion is strengthened by the provision of compensation to the individual whose business is affected by an order prohibiting the sale of milk, for were his farm certainly the source of contagion no compensation should be due. The enunciation and adoption of this principle by the court is of considerable significance. Particularly is it to be noted that though there was no proof of disease in the cows, nor of any case of illness occurring at the farm, nor of any pollution of the water supply, the sheriff used his authority to stop the supply of milk. Speaking in his judicial capacity he held it was sufficient to establish a case for reasonable suspicion, and such suspicion he considered had an abundant basis in the fact that almost all those affected with the disease had this common milk supply. That was sufficient to justify a reasonable belief that the milk supply was the source of contamination, and this being established no further evidence was necessary. Thus the result of the case was a triumph for the burgh health authorities, and, what is more important, for the preservation of the public health. It can hardly be a mere coincidence that very soon after the sheriff's order the excessive prevalence of enteric subsided and that in two or three weeks the reported cases did not exceed the usual average of the district.

